# Supplementary material for: Directed migration shapes cooperation in spatial ecological public goods games
Source: PLoS Comput Biol. 2019 Aug 8;15(8):e1006948. doi: 10.1371/journal.pcbi.1006948 (PMC6687102; doi:10.1371/journal.pcbi.1006948)
Supplement: S1 Appendix — (PDF) [file pcbi.1006948.s001.pdf]

Specifics of Ecological Public Goods Dynamics

As introduced in the main text, the interplay of ecological and evolutionary dynamics is modelled by

$$\partial_t u = u\left[w(b + f_C) - d\right] \tag{S1.1a}$$

$$\partial_t v = \underbrace{v\left[w(b + f_D) - d\right]}_{\text{ecological dynamics}}. \tag{S1.1b}$$

where $w = 1 - u - v$ reflects reproductive opportunities that diminish for increasing population densities [1]. While the death rate, d, remains constant, the effective birth rates of cooperators, $w(b + f_C)$, and defectors, $w(b + f_D)$, are determined by the average payoffs

$$f_D = r\frac{u}{u+v}\left(1 - \frac{1 - w^N}{(u+v)N}\right)c, \tag{S1.2a}$$

$$f_C = f_D - \left(1 + (r-1)w^{N-1} - r\frac{1 - w^N}{(u+v)N}\right)c \tag{S1.2b}$$

plus the baseline birth rate, b, because in the limit $v \to 1$ the payoff f_C can become negative, which is biologically not meaningful [1]. The ecological dynamics results in variable interaction group sizes with an effective, expected group size of $S = (u + v)N$. Thus, as long as $S > r$, defection dominates, the public resource is overexploited and the population density declines. Consequently, interactions occur in smaller groups and returns are split among fewer individuals. For sufficiently small groups, $S < r$, cooperation becomes favourable again, the population density recovers, S increases and the cycle continues.

References

1. Hauert C, Yuichiro Wakano J, Doebeli M. Ecological Public Goods Games: cooperation and bifurcation. Theoretical Population Biology. 2008;73:257–263.
