# Supplementary material for: Directed migration shapes cooperation in spatial ecological public goods games
Source: PLoS Comput Biol. 2019 Aug 8;15(8):e1006948. doi: 10.1371/journal.pcbi.1006948 (PMC6687102; doi:10.1371/journal.pcbi.1006948)
Supplement: S2 Appendix — (PDF) [file pcbi.1006948.s002.pdf]

Derivation of Directed Migration Terms

The directed migration terms $\mp K\nabla\cdot(w\phi\nabla\psi)$ can be derived as the continuum limit of an intuitive difference equation. For simplicity and accessibility we restrict the detailed derivation to a single spatial dimension with periodic boundary conditions and then outline extensions to higher dimensions.

Difference Equation

Let ϕ_f^t be the normalized density of strategy ϕ at time t found at focal site f. The attraction of strategy $\phi = u$ (or v) to cooperators u is determined by:

(i) If the cooperator density u_f^t at the focal site f is higher than at adjacent sites $u_{f\pm h}^t$, a proportion of type $\phi_{f\pm h}^t$ migrates to the focal site. This migration is moderated by the reproductive opportunities at the focal site, w_f^t.

(ii) Conversely, if densities of cooperators are higher at adjacent sites $u_{f\pm h}^t$, a proportion of type ϕ_f^t migrates from the focal site to the adjacent site(s), again subject to the respective reproductive opportunities $w_{f\pm h}^t$.

(iii) The sensitivity to density differences may be different for cooperators and defectors. The parameters A_C and A_D determine their respective migration rate.

The difference in cooperator densities at the focal and adjacent sites determines the direction of the flux of strategy ϕ_f^t at the focal site f. This conditionality is conveniently captured by the indicator $\mathbf{1}_{\{a<b\}}$, which evaluates to 1 if $a < b$ and to 0 otherwise. Thus, the net migration for strategy ϕ_f^t at the focal site between time t and $t + \Delta t$ due to attraction to cooperators is given by:

$$\underline{\phi_f^{t+\Delta t} - \phi_f^t}\ \underline{\frac{\phi_f^{t+\Delta t} - \phi_f^t}{\Delta t}} = A \cdot \left(\sum_{i\in\{f+h,f-h\}} (u_f^t - u_i^t) \cdot \left(\mathbf{1}_{\{u_f^t < u_i^t\}} w_i^t \phi_f^t + \mathbf{1}_{\{u_f^t \geq u_i^t\}} w_f^t \phi_i^t \right) \right), \tag{S1.1}$$

where A refers to the strength of attraction with $A = A_C$ for $\phi = u$ and $A = A_D$ for $\phi = v$, respectively. An analogous argument yields the net migration due to the repulsion from defectors:

$$\phi_f^{t+\Delta t} - \phi_f^t \frac{\phi_f^{t+\Delta t} - \phi_f^t}{\Delta t} = -R \cdot \left(\sum_{i \in \{f+h, f-h\}} (v_f^t - v_i^t) \cdot \left(\mathbf{1}_{\{u_f^t < u_i^t\}} w_i^t \phi_f^t + \mathbf{1}_{\{u_f^t \geq u_i^t\}} w_f^t \phi_i^t \right) \right), \tag{S1.2}$$

where R indicates the strength of repulsion with $R = R_C$ for $\phi = u$ and $R = R_D$ for $\phi = v$, respectively.

Continuum Limit

In the continuum limit we let the spatial distance between adjacent sites, h, and the time increment, Δt, approach zero. Hence, h and Δt refer to infinitesimally small quantities. Let us first focus on the right hand side of Eq. (S1.1). Taylor expansion yields

$$\begin{aligned} u_{f\pm h} &\approx u_f \pm h \cdot u'_f + h^2/2 \cdot u''_f, \\ \phi_{f\pm h} &\approx \phi_f \pm h \cdot \phi'_f, \\ w_{f\pm h} &\approx w_f \pm h \cdot w'_f \end{aligned}$$

where $'$'s indicate spatial derivatives and the superscript t for the time has been omitted. Using

$$\begin{aligned} u_f - u_{f\pm h} &\approx h \cdot \left(\mp u'_f - h/2 \cdot u''_f \right), \\ w_{f\pm h}\phi_f &\approx w_f \phi_f \pm h w'_f \phi_f, \\ w_f \phi_{f\pm h} &\approx w_f \phi_f \pm h w_f \phi'_f, \end{aligned}$$

the summation in Eq. (S1.1) runs over

$$h \cdot \left(\mp u'_f - h/2 \cdot u''_f \right) \cdot \left(\mathbf{1}_{\{u_f < u_{f\pm h}\}} (w_f \phi_f \pm h w'_f \phi_f) + \mathbf{1}_{\{u_f \geq u_{f\pm h}\}} (w_f \phi_f \pm h w_f \phi'_f) \right).$$

Note that $w_f \phi_f$ appears in the two complementary indicators and hence simplifies to

$$\mp h u'_f w_f \phi_f - h^2 \cdot \left(\frac{1}{2} u''_f w_f \phi_f + \mathbf{1}_{\{u_f < u_{f \pm h}\}} u'_f w'_f \phi_f + \mathbf{1}_{\{u_f \geq u_{f \pm h}\}} u'_f w_f \phi'_f \right).$$

Adding contributions from $f \pm h$ cancels terms of order h and yields

$$-h^2 \cdot \left(u''_f w_f \phi_f + u'_f \cdot \sum_{i \in \{f+h, f-h\}} \mathbf{1}_{\{u_f < u_i\}} w'_f \phi_f + \mathbf{1}_{\{u_f \geq u_i\}} w_f \phi'_f \right). \tag{S1.3}$$

Note that the terms in parentheses in Eq. (S1.3) resemble $u''_f w_f \phi_f + u'_f (w'_f \phi_f + w_f \phi'_f) = (w_f \phi_f u'_f)'$ when disregarding the sum and indicators. If u_f is on a slope, e.g. $u_{f-h} < u_f < u_{f+h}$ it is easy to see that equality holds. However, if the cooperator density is at a local maximum or minimum at the focal site f, i.e. $u_{f-h} < u_f > u_{f+h}$ or $u_{f-h} > u_f < u_{f+h}$, then one of the terms $w'_f \phi_f$ or $w_f \phi'_f$ cancels while the other is doubled. Nevertheless the resulting error is negligible because both terms are multiplied by u'_f which is ≈ 0 in the vicinity of an extremum.

Using Taylor expansion of the left-hand-side of Eq. (S1.1), $\phi_f^{t+\Delta t} - \phi_f^t \approx \Delta t \cdot \partial_t \phi_f$ and an analogous calculation for Eq. (S1.2) yields The continuum limit, $\Delta t, h \to 0$ with $h^2/\Delta t \to 1$, of Eqs. (S1.1) and (S1.2) yields the dynamical equations just based on directed migration:

$$\partial_t \phi = -A \partial_x (\phi w \cdot \partial_x u), \tag{S1.4a}$$

$$\partial_t \phi = +R \partial_x (\phi w \cdot \partial_x v),. \tag{S1.4b}$$

in the limit $h \to 0$ and $\Delta t \to 0$ with $h^2/\Delta t \to 1$.

In higher spatial dimensions the above derivation applies to each spatial dimension separately and yields

$$\partial_t \phi = \mp K (\partial_x (\phi w \partial_x \psi) + \partial_y (\phi w \partial_y \psi)) = \mp K \nabla \cdot (\phi w \nabla \psi) \tag{S1.5}$$

for two dimensions.
