# Supplementary material for: Directed migration shapes cooperation in spatial ecological public goods games
Source: PLoS Comput Biol. 2019 Aug 8;15(8):e1006948. doi: 10.1371/journal.pcbi.1006948 (PMC6687102; doi:10.1371/journal.pcbi.1006948)
Supplement: S3 Appendix — (PDF) [file pcbi.1006948.s003.pdf]

Pattern Formation

Dispersion Relation

Homogeneous coexistence with a density distribution according to the well-mixed equilibrium Q (see Eq. (1)) is a trivial steady state of the selection–migration dynamics, Eq. (3). However, even if Q is stable, the homogeneous distribution may not be stable to spatial perturbations. A small perturbation vector $p(t,x,y) = \epsilon(\delta u \exp(ik(x+y)), \delta v \exp(il(x+y)))$ with an amplitude of $\epsilon \ll 1$ and mode k for cooperators and mode l for defectors grows or contracts with $\delta u \sim \exp(\lambda(k,l)t)$ and $\delta v \sim \exp(\lambda(k,l)t)$. The spatial dynamics in the vicinity of Q in response to the perturbation are captured by

$$\begin{pmatrix} \partial_t \hat{u} \\ \partial_t \hat{v} \end{pmatrix} = \left(J_I + \begin{pmatrix} k^2 & 0 \\ 0 & l^2 \end{pmatrix} \cdot J_S \right) \cdot \begin{pmatrix} \hat{u} \\ \hat{v} \end{pmatrix}. \tag{S3.1}$$

The largest real part of the eigenvalues of Eq. (S3.1) is a function of k and l and called the dispersion relation, $\lambda(k,l)$. Any mode with $\lambda(k,l) > 0$ is amplified, while those with $\lambda(k,l) < 0$ are dampened. If $\lambda(k,l) < 0 \ \forall k,l$ the homogeneous state is stable.

The most unstable mode (k_*, l_*) is given by $\max \lambda(k,l) = \lambda(k_*, l_*) > 0$ and is particularly important because it dominates all other unstable modes due to its exponential amplification. The characteristic length scale of the resulting density pattern is inversely proportional to k_* for cooperators and to l_* for defectors. Numerical observations indicate that these characteristic length scales match closely, $k_* \approx l_*$, due to the dependence of defectors on cooperators for survival (as $d > b$). Therefore, we restrict the analysis to perturbations with dependent wavelengths, $k = l$, and abbreviate $\lambda(k) = \lambda(k,l)$. Note that $\lambda(0) > 0$ iff Q is unstable, i.e. for $r < r_{\text{Hopf}}$, and hence $k = 0$ is referred to as the temporal mode because it only triggers changes over time. If $\lambda(0) > 0$ and $\lambda(k_*) > 0$ for some $k_* > 0$, temporal and spatial instabilities interfere and can

	min A_C	max A_D	max R_C	min R_D
analytics	0.24	0.81	0.74	1.09
numerics	0.26	0.76	0.68	1.2

Table A. Thresholds for pattern formation. Quantitative comparison of analytical predictions from the dispersion relation and from numerical integration for the onset of pattern formation. For activating directed migration A_C, R_D the threshold marks a lower bound but an upper bound for inhibiting directed migration A_D, R_C. Numerical thresholds systematically deviate from analytical predictions by requiring higher (lower) directed migration rates for the onset (suppression) of patterns. This difference is expected to decrease when increasing integration times. *Parameters* as in Fig. 1 but with $D_D = 0.5$ for A_C, R_D and $D_D = 0.7$ for A_D and R_C and $t = 2000$. Initial configuration: homogeneous densities Q perturbed by Gaussian noise with standard deviation 0.01.

result in dynamical patterns. For $k_* \to \infty$, the emerging patterns have a characteristic length of 0, which implies that no spatial discretization is fine enough to capture them. The asymptotic behaviour of $\lambda(k)$ for $k \to \infty$ is determined by J_S, Eq. (5b). More specifically, $\det(J_S) > 0$ and $\operatorname{tr}(J_S) < 0$ ensure that the largest eigenvalue has negative real part such that $\lambda(k) < 0$ for $k \to \infty$. This is the case if the diffusion of cooperators outweighs their aggregation, $D_C > A_C u_{\mathrm{eq}} w_{\mathrm{eq}}$, at the homogeneous density Q.

The impact of separate increases in migration rates A_C, A_D, R_C, R_D on the dispersion relation is illustrated in S1 Figure. Increasing cooperator aggregation, A_C, and spreading of defectors, R_D, increases $\lambda(k)$ and renders intermediate modes unstable. The dominant mode k_* increases with A_C, which reduces the characteristic length of patterns. Conversely, increasing R_D decreases k_* and increases the characteristic length scale. In contrast, increasing cooperator flight, R_C, and hunting by defectors, A_D, reduces $\lambda(k)$ and eliminates unstable modes. The thresholds for pattern formation predicted by the dispersion relation agree well with numerical integration, see Table A in S3 Appendix.

Necessary Criteria

Turing [1] famously derived the necessary criteria for pattern formation in reaction-diffusion systems. Thereafter, Segel and Jackson [2] refined Turing's work to determine requirements on the diffusion rates which facilitate pattern formation in activator–inhibitor systems. Wakano et al. [3] translated these requirements into the context of cooperation in spatial public goods games. Here we extend the latter analysis to the selection-migration model which includes directed migration.

Pattern formation is driven by the interplay of activators (cooperators) and inhibitors (defectors). Close to the equilibrium Q this implies that an increase in cooperation triggers an increase in cooperator and defector densities, while an increase in defection results in a decrease of both types. Consequently, $a_{CC} > 0$, $a_{DC} > 0$ and $a_{CD} < 0$, $a_{DD} < 0$ in the Jacobian J_I, see Eq. (5a). In addition, we require that Q is stable, which translates to

$$\mathrm{tr}(J_I) = a_{CC} + a_{DD} < 0 \tag{S3.2}$$

$$\det(J_I) = a_{CC}a_{DD} - a_{CD}a_{DC} > 0. \tag{S3.3}$$

An equivalent requirement is $r > r_{\mathrm{Hopf}}$ with r sufficiently close to r_{Hopf} to ensure that Q is a focus and the activator–inhibitor relation of cooperators and defectors is maintained. From the stability of Q follows that the temporal mode is stable, $\lambda(0) < 0$. Additionally, we require $\lambda(k) < 0$ for $k \to \infty$ and thus that the entries of the Jacobian J_S, see Eq. (5b), satisfy $s_{CC} < 0$, $s_{CD} < 0$ and $s_{DC} > 0$, $s_{DD} < 0$ or, equivalently, $D_C > A_C u_{\mathrm{eq}} w_{\mathrm{eq}}$.

Since $\mathrm{tr}(J_I + k^2 J_S) = a_{CC} + a_{DD} + k^2(s_{CC} + s_{DD}) < 0$ the stability condition for the homogeneous distribution with densities according to Q reduces to $\det(J_I + k^2 J_S) > 0$. More specifically,

$$\begin{aligned}\det(J_I + k^2 J_S) &= (a_{CC} + k^2 s_{CC})(a_{DD} + k^2 s_{DD}) - (a_{CD} + k^2 s_{CD})(a_{DC} + k^2 s_{DC}) \\ &= \det(J_I) + k^4 \det(J_S) + k^2 P,\end{aligned}$$

with $P = a_{CC}s_{DD} + a_{DD}s_{CC} - a_{CD}s_{DC} - a_{DC}s_{CD}$. Since $\det(J_I) > 0$ and $\det(J_S) > 0$ intermediate modes can only become unstable if $P < 0$, which requires

$$\underbrace{(-a_{DD})(A_C u_{\mathrm{eq}} w_{\mathrm{eq}} - D_C) - a_{DC} R_C u_{\mathrm{eq}} w_{\mathrm{eq}}}_{\substack{\text{activation through} \\ \text{cooperator migration}}} > \underbrace{(-a_{CD}) A_D v_{\mathrm{eq}} w_{\mathrm{eq}} + a_{CC}(-R_D v_{\mathrm{eq}} w_{\mathrm{eq}} - D_D)}_{\substack{\text{inhibition through} \\ \text{defector migration}}}. \tag{S3.4}$$

Thus, the activating effects of cooperator migration must exceed the inhibitory effect of defector migration. Consequently, pattern formation can be promoted by increasing aggregation rates of cooperators, A_C, spreading of defectors, R_D, or diffusion of

defectors, D_D, as well as by reducing hunting rates of defectors, A_D, fleeing of cooperators, R_C, or diffusion of cooperators, D_C. The reverse suppresses patterns and stabilizes homogeneous distributions.

While Eq. (S3.4) is a necessary condition it is not sufficient because, in addition, $\det(J_I + k^2 J_S) < 0$ must hold at its minimum, k_*, which is given by

$$k_*^2 = -\frac{P}{2\det(J_S)}.$$

At k_* the stability condition $\det(J_I + k^2 J_S) < 0$ simplifies to

$$P < -2\sqrt{\det(J_I)\det(J_S)}. \tag{S3.5}$$

Effectively, this increases the threshold for pattern formation of Eq. (S3.4) by adding $\sqrt{\det(J_I)\det(J_S)}$ to the right hand side. The factor 2 cancels with the factor 2 found in the individual components of J_S, Eq. (5b).

Unfortunately, however, the magnitude of this increase sensitively depends on game parameters as well as migration rates and thus largely eludes intuitive interpretations. Nevertheless, for the impact of migration we have

$$\det(J_S) = (D_C - A_C u_{\mathrm{eq}} w_{\mathrm{eq}})(R_D v_{\mathrm{eq}} w_{\mathrm{eq}} + D_D) + u_{\mathrm{eq}} v_{\mathrm{eq}} w_{\mathrm{eq}}^2 A_D R_C, \tag{S3.6}$$

which means that increases in directed and undirected migration rates all increase the threshold with the exception of increases in cooperator aggregation, A_C.

In summary, our analysis is based on the following three assumptions:

1. Q is stable (i.e. $r > r_{\mathrm{Hopf}}$),
2. $\lambda(k) < 0$ for $k \to \inf$,
3. $\det(J_I + k^2 J_S) > 0$ for some $k > 0$.

Note that condition 3 is essential for unstable stable modes to induce pattern formation, while conditions 1 and 2 are likely more conservative than necessary. More specifically, the formal approach to determine conditions Turing instabilities requires that Q is stable (condition 1). However, numerical results indicate that the criterion maintains

relevance for $r < r_{\mathrm{Hopf}}$ and was termed diffusion-induced co-existence as opposed to diffusion induced instability for $r > r_{\mathrm{Hopf}}$ [3]. Because of the unstable temporal mode emerging patterns may not be static but rather change intermittently or even exhibit spatio-temporal chaos. The primary purpose of condition 2 is to prevent the amplification of high-frequency perturbations because this inevitably interferes with any discretization chosen to numerically integrate the time evolution of cooperator and defector densities (see Eq. (3) in main text). Therefore, numerical solutions to the migration-selection system are unlikely to maintain the basic requirements of smoothness and robustness. We also note that for a horizontal asymptote, with $0 < \lambda(k) < \lambda(k_*)$ for $k \to \infty$, the instability of high frequency modes may not matter because of the exponentially faster amplification of the dominant mode k_*. This scenario only arises for parameters satisfying $D_C \approx A_C u_{\mathrm{eq}} w_{\mathrm{eq}}$, i.e. for similar cooperator aggregation and diffusion. However, in this parameter region numerical challenges start to arise, which are likely related to the instability of high frequency modes. Hence we restricted the analysis to the more conservative condition 2.

References

1. Turing AM. The Chemical Basis of Morphogenesis. Philosophical Transactions of the Royal Society B. 1952;237(641):37–72.

2. Segel LA, Jackson JL. Dissipative structure: an explanation and an ecological example. Journal of theoretical biology. 1972;37(3):545–559.

3. Wakano JY, Nowak MA, Hauert C. Spatial dynamics of ecological public goods. Proceedings of the National Academy of Sciences. 2009;106(19):7910–7914.
