# Supplementary figures and images for: Directed migration shapes cooperation in spatial ecological public goods games
Source: PLoS Comput Biol. 2019 Aug 8;15(8):e1006948. doi: 10.1371/journal.pcbi.1006948 (PMC6687102; doi:10.1371/journal.pcbi.1006948)

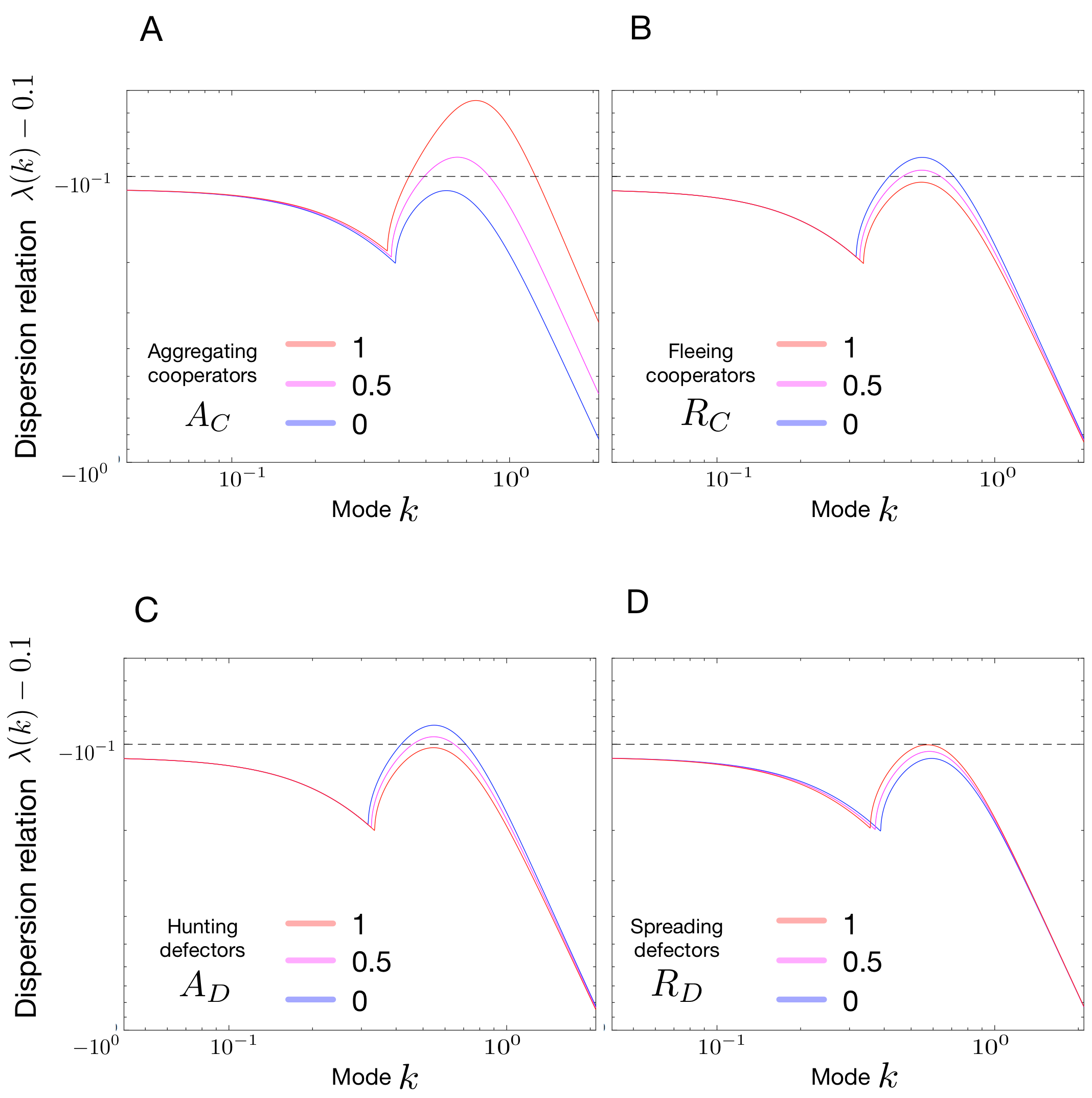

Supplement: S1 Fig — (A) aggregating cooperators, AC, and (D) spreading defectors, RD, have both the potential to cause spatial instabilities through increased migration. In the process, the dominant mode k* increases with increased aggregating cooperators, AC, and decreases with spreading defectors, RD. In contrast, (C) hunting defectors AD and (D) fleeing cooperators RC stabilize the system. Parameters: 2.4 = r > rHopf (such that λ(0) < 0), as well as DC > ACueqweq (to ensure λ(k) < 0 for k → ∞). (A), (D) DD = 0.5, (B), (C) DD = 0.7. Other parameters as in Fig 1. (TIFF) [file pcbi.1006948.s004.tiff]

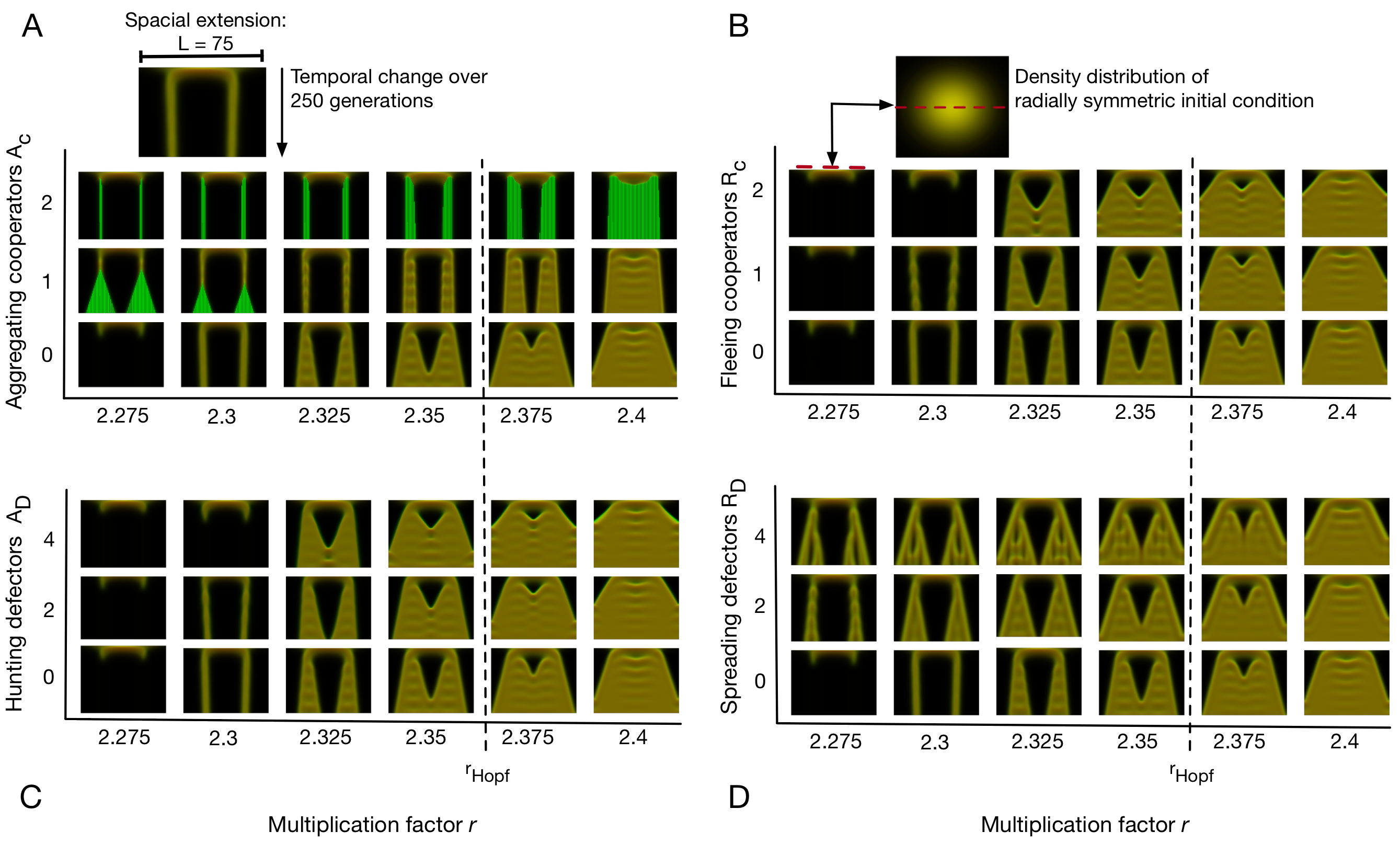

Supplement: S2 Fig — Each panel illustrates the dynamics for a single type of directed migration as a function of the multiplication factor r in the proximity of rHopf. The small rectangles depict the cross section of the density distribution through the middle of the square L × L domain as a function of time from top to bottom. The color brightness indicates the density of cooperation (green) and defection (red) with coexistence (yellow) and vacant space (black). (A) Aggregating cooperators oppose the effects of diffusion and hence slow down the population expansion. The positive feedback between cooperator densities and aggregation can cause discontinuous distributions and the breakdown of numerical methods. (B) Hunting defectors increase competition in areas where cooperators concentrate. This allows cooperators to escape into unpopulated terrain but also increases the risk of extinction. (C) Fleeing cooperators avoid defectors and hence readily explore vacant space but with the defectors at their tails also tend to spread themselves too thin and risk extinction. (D) Spreading defectors again promote pattern formation and thereby support the survival of the population. Parameters: same as in Fig 1 but with Gaussian initial condition without noise and t = 250. (TIFF) [file pcbi.1006948.s005.tiff]

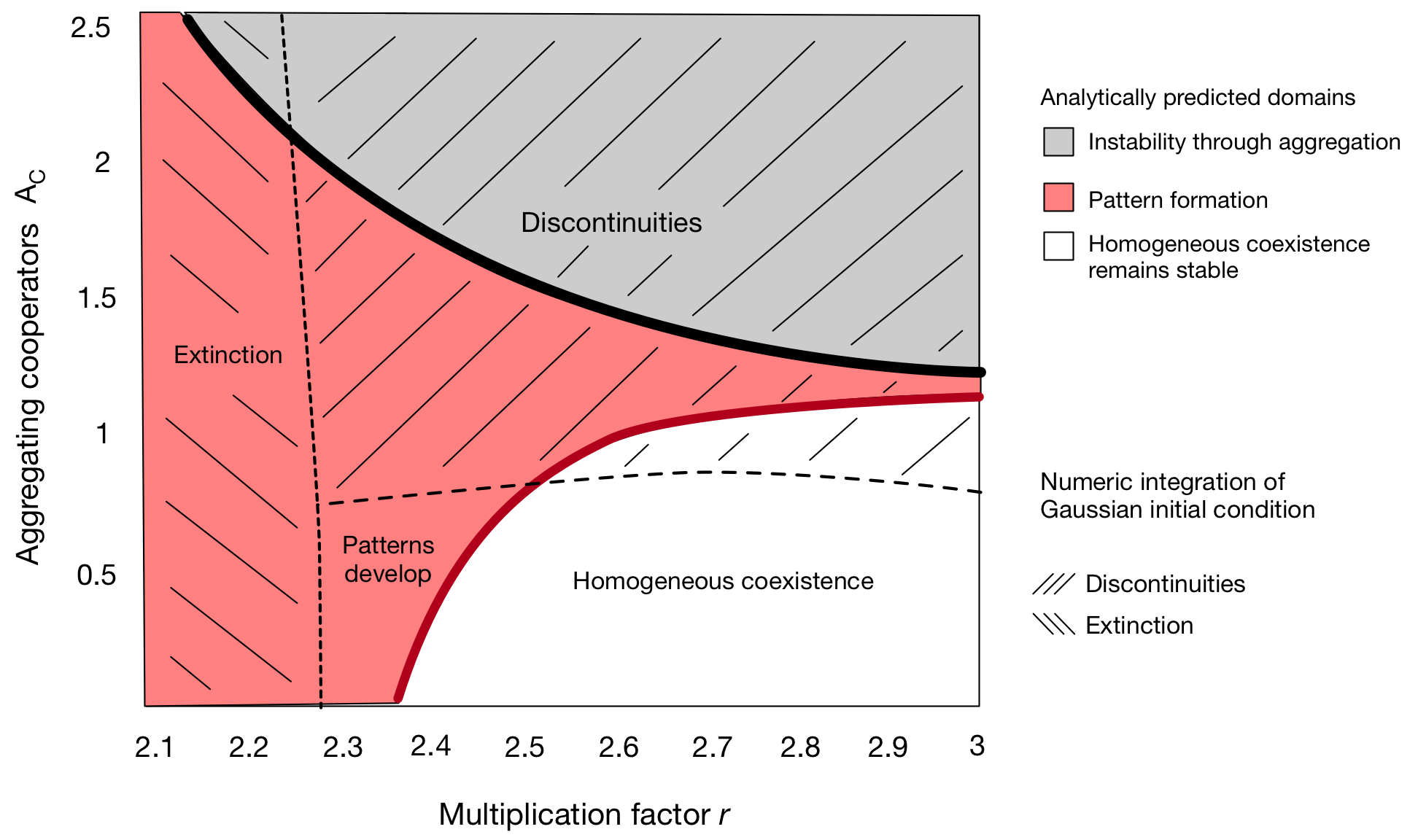

Supplement: S3 Fig — Analytical findings distinguish three domains based on AC and the multiplication factor r: (i) aggregation is sufficiently strong to destabilize the homogeneous equilibrium (grey shaded region, ACueqweq > DC); (ii) unstable modes give rise to pattern formation (red shaded region, see Eq. (S4) in S3 Appendix); (iii) stable homogeneous coexistence (unshaded region). However, initial conditions significantly impact the emerging dynamics. Heterogeneous initial distributions trigger migration and aggregation of cooperators. Discontinuities emerge by t = 250 from a Gaussian initial distribution when the selection-diffusion system is numerically integrated (above dashed horizontal line). Below the dashed horizontal-line, patterns emerge (in red shaded region) or homogeneous coexistence is regained (in unshaded region) as predicted. For small r heterogeneous distributions are unable to develop and the population goes extinct (left of dashed vertical line). Parameters: same as in S2 Fig, except DD = 0.5 to promote smooth patterns. (TIFF) [file pcbi.1006948.s006.tiff]
